# Antibiotic prescriptions to preschool children with respiratory tract infections in primary healthcare

**DOI:** 10.1093/jacamr/dlaf231

**Published:** 2026-01-08

**Authors:** Therese Renaa, Louise Emilsson, Sigurd Høye, Marius Skow, Guro H Fossum

**Affiliations:** Department of General Practice, The Antibiotic Centre for Primary Care, Institute of Health and Society, University of Oslo, Oslo, Norway; Department of General Practice, General Practice Research Unit (AFE), Institute of Health and Society, University of Oslo, Oslo, Norway; Vårdcentralen Värmlands Nysäter and Centre for Clinical Research, County Council of Värmland, Värmlands Nysäter, Karlstad 661 95, Sweden; Department of Medical Epidemiology and Biostatistics, Karolinska Institutet, Stockholm, Sweden; School of Medical Science, University of Örebro, Örebro, Sweden; Department of General Practice, The Antibiotic Centre for Primary Care, Institute of Health and Society, University of Oslo, Oslo, Norway; Department of General Practice, The Antibiotic Centre for Primary Care, Institute of Health and Society, University of Oslo, Oslo, Norway; Department of General Practice, The Antibiotic Centre for Primary Care, Institute of Health and Society, University of Oslo, Oslo, Norway; Department of General Practice, General Practice Research Unit (AFE), Institute of Health and Society, University of Oslo, Oslo, Norway

## Abstract

**Background:**

Correct use of antibiotics ensures necessary treatment for patients while antibiotic resistance is reduced. Respiratory tract infections (RTIs) are common in preschool children. Young children receive a large proportion of the total amount of antibiotics, and also in low-prescribing countries such as Norway.

**Objectives:**

Explore the contacts, rate of antibiotic prescriptions and choice of antibiotics in the treatment of RTIs in preschool children in general practice from 2012 to 2019. Methods Descriptive registry study on complete population data of antibiotic prescriptions administered to Norwegian pre-school children with RTIs, in the period 2012 – 2019, after consultations with a general practitioner

**Results:**

The total prescription rate was reduced from 28% in 2012 to 19% in 2019. There were small yearly variations in prescription rates. Most antibiotics were prescribed to 1- and 2-year-olds. Upper RTI was the most used diagnosis and accounted for 25% of the total amount of antibiotics prescribed.

Total RTI episode rate was 941 episodes/1000 children in 2012, reduced by 17% to 2019 when there were 777 episodes/1000 children. The reduction in antibiotic prescription to children with otitis was associated with a decline in episode rate.

More than 81% of prescribed antibiotics were penicillins, only 16% were macrolides and 3% were other antibiotics. The use of phenoxymethylpenicillin increased in the period from 50% in 2012 to 68% in 2019.

**Conclusions:**

There is room for improvement in adherence to guidelines and antibiotic stewardship also in low-prescribing countries. Antibiotic prescribing is closely linked to prescription rates and health-seeking behaviours, offering valuable insights for targeted antibiotic stewardship campaigns.

## Introduction

Antimicrobial resistance (AMR) is a threat to modern medicine. Appropriate use of antibiotics is imperative to preserve the efficacy of the treatment. There is a strong correlation between the total amount of antibiotics used in a population and the level of AMR.^1^ Most antibiotics are prescribed in primary healthcare.^2^ Norway has a relatively low total prescription of antibiotics. However, there is still room for improvement in Norway.^3^ The first Norwegian action plan against antimicrobial resistance dates to the period 2000–2004, followed by an integrated national strategy in the period 2008–2012.^4^ In 2015 the next national strategy was launched, with a defined target to reduce human antibiotic use by 30% from 2015 to 2019.^5^ The goal was reached after the strategy period was prolonged to 2024 because of the COVID-19 pandemic. There are national guidelines for antibiotic treatment in general practice, with narrow spectrum penicillin and phenoxymethylpenicillin the drug of choice for respiratory tract infections (RTIs).^6^ The new national strategy aims to reduce antibiotic consumption 10% from 2024 to 2033 to combat AMR.^7^

Children are exposed to a wide variety of infections during the first years of life, most often self-limiting viral RTIs.^8^ National guidelines for antibiotic treatment in primary healthcare give recommendations for appropriate use of antibiotics to children with RTIs,^6^ but there is reason to believe that the guidelines are not always followed.^9^ Earlier studies indicate widespread inappropriate antibiotic prescription to children with RTIs.^10,11^ Preschool children receive more antibiotics than other age groups.^2,12,13^ Unnecessary use of antibiotics in early life may be associated with higher risk for unwanted consequences. A Scandinavian cohort study showed that antibiotic use in early life was associated with development of inflammatory bowel disease in childhood and adolescence.^14^ There are also studies investigating the association with allergy and asthma.^15^ Knowledge of the antibiotic prescription patterns and health-seeking behaviour in a population identifies areas for improvement and target antibiotic stewardship interventions. The Norwegian national health registries give a complete overview of the use of health services and offer a unique population-level insight into the use of healthcare services and prescription medication, allowing us to study preschool children with RTIs.

In this study, we aim to explore the contacts, rate of antibiotic prescriptions and choice of antibiotics in the treatment of RTIs in preschool children in general practice from 2012–2019. The COVID-19 pandemic altered health-seeking behaviours between 2020 and 2022. Recent prescription data indicates that antibiotic usage in the post-pandemic years is comparable to the levels seen in 2019. We believe that the trends observed from 2012 to 2019 provide valuable insights that are relevant for future stewardship campaigns.

## Materials and methods

### Ethics

The Regional Committee for Medical and Health Research Ethics, REC Southeast (ref. 2016/2283), and the Norwegian Data Protection Authority (ref. 282558) approved the study design and data treatment. The research was conducted in accordance with the Declaration of Helsinki and institutional standards.

### Design

This study is a descriptive analysis based on observational data collected from national health registries between 2012 and 2019. Norway’s healthcare system is publicly funded and universally accessible, with every resident registered with a general practitioner (GP). The financing system mandates that all healthcare interactions are recorded in national registries.

### Data sources

The data were combined from four different health registries: KUHR (The Norwegian control and reimbursement registry), SSB (Norwegian statistics), NPR (The National Patient Registry) and NorPD (the Norwegian prescription registry). Collectively, these registries provide comprehensive information on healthcare contacts across the entire population.

KUHR gives information on health contacts with primary healthcare providers in Norway. The data collected provide information on the patient’s diagnosis according to International Classification of Primary Care (ICPC-2).^16^ We included regular and out-of-hours GP consultations in the study. The SSB provides information on age and gender.

The NPR is the register of all activity generated in hospitals. The prescription register (NorPD) gives information on all prescriptions dispensed from pharmacies, classified according to the Anatomical Therapeutic Chemical classification (ATC),^17^ with dates of collection of prescription, quantity, and duration. We used NPR to exclude children admitted to hospital during their first contact with a GP.

The data were combined by using unique encrypted personal identification numbers.

### Population

The material includes children between the age of 1 and 5 years in each year of the study period from 2012–2019. Infants under 1 year were excluded due to the complexities in diagnosing and treating infections in this age group, which often leads to higher hospitalization rates and a greater reliance on symptom-based diagnoses. In addition, we excluded children who were admitted to hospital or specialist on the same day as the first contact in an episode. Each individual child may have several contacts in the study period.

### Diagnoses

**Figure 1.**
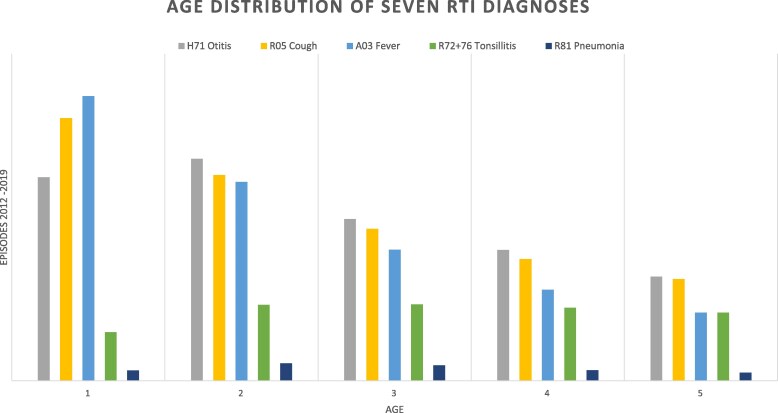
Age distribution of mean RTI episodes/1000 children 1–5 years old.

**Figure 2.**
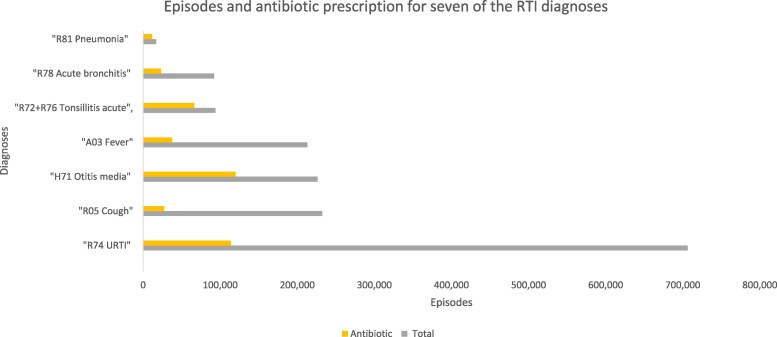
RTI episodes and RTI episodes with antibiotic prescription for the seven diagnoses with the highest antibiotic prescription rates.

**Figure 3.**
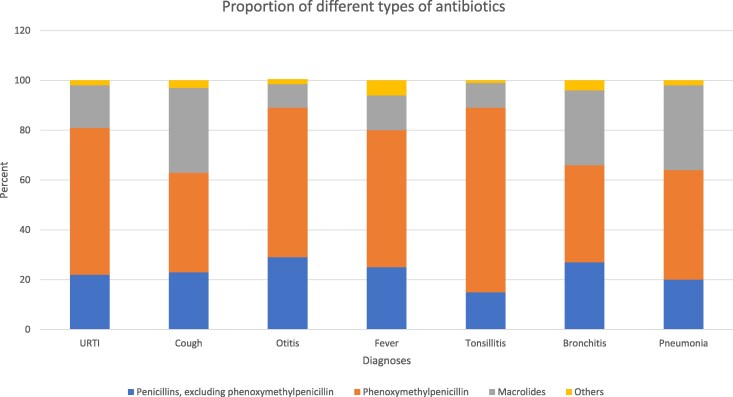
Choice of antibiotics for the seven RTI diagnoses with highest total amount of antibiotics prescribed to children 1–5 years.

Diagnoses were defined using ICPC-2. The material includes contacts with diagnostic codes for respiratory symptoms and infections (R01—R09, R21, R23, R25, R29, R71–R83), ear pain and otitis (H01, H71), fever (A03), infection (A78) and viral infection (A76, A77). All diagnoses are listed in Supplementary (Table S1, available as Supplementary data at *JAC-AMR* Online). We have chosen to present seven diagnoses in more detail in Tables 1 and 2, and in Figures 1–3: ‘R05 Cough’, R74 URTI’, ‘A03 Fever’ and ‘R78 Bronchitis/bronchiolitis’, ‘H71 Otitis’, ‘R72+76 Strep throat + Acute tonsillitis’ and ‘R81 Pneumonia’. These were chosen for their clinical significance and in sum they represent 88% of the total amount of antibiotics prescribed to children aged 1–5 with RTI.

**Table 1.** Acute respiratory tract (RTI) episodes in children 1–5 years in Norwegian general practice 2012–2019, with episode rate for seven diagnoses with high antibiotic use

	Total	Boys	Girls
Patients (%)	578 965	301 783 (52)	277 182 (48)
Mean age in years at first contact (SD)	1.9 (1.3)	1.9 (1.3)	2.0 (1.3)
RTI contacts (%)	3 126 818	1 695 302 (54)	1 431 516 (46)
RTI episodes (%)	2 003 904	1 073 326 (54)	930 578 (46)
Mean contacts per episode (SD)	2.3 (1.7)	2.3 (1.8)	2.2 (1.9)
Rate episodes			
RTI episodes/1000 children/year	811	846	774
Age			
1 year	1 279	1 358	1 202
2 years	1 073	1 127	1 023
3 years	747	781	716
4 years	557	574	544
5 years	458	453	441
7 diagnoses with high antibiotic use			
URTI (R74)	287	296	277
Cough (R05)	94	95	92
Otitis (H71)	91	96	86
Fever (A03)	86	87	86
Tonsillitis (R72 + 76)	38	41	35
Bronchitis (R78)	37	40	34
Pneumonia (R81)	7	7	7

**Table 2.** Episode rates, antibiotic prescription rates and proportion of phenoxymethylpenicillin for the seven RTI diagnoses with highest amount of antibiotics prescribed 2012–2019 for children 1–5 years old in Norwegian primary healthcare

	Total	2012	2013	2014	2015	2016	2017	2018	2019	Linear regression Mean annual change (95%CI)
All RTIs										
Episodes/1000 children	811	941	827	815	772	814	785	757	777	−18 (−33, −4)
Antibiotic prescriptions/100 episodes	23	28	24	25	23	22	20	19	19	−1 (−1, −1)
Phenoxymethylpenicillin/100 antibiotic prescriptions	57	50	53	54	56	60	63	65	68	3 (2,3)
URTI										
Episodes/1000 children	287	291	267	273	268	297	294	293	310	4(0,9)
Antibiotic prescriptions/100 episodes	16	20	18	19	17	15	14	13	13	−1 (−1, −1)
Phenoxymethylpenicillin/100 antibiotic prescriptions	59	52	55	55	58	60	63	65	69	2 (2,3)
Cough										
Episodes/1000 children	94	136	103	104	89	87	86	75	71	−8 (−11, −4)
Antibiotic prescriptions/100 episodes	12	16	12	13	11	11	10	9	9	−1 (−1, −1)
Phenoxymethylpenicillin/100 antibiotic prescriptions	40	33	38	40	41	45	44	46	49	2 (2,3)
Otitis										
Episodes/1000 children	91	117	102	101	91	89	81	76	74	−5 (−7, −3)
Antibiotic prescriptions/100 episodes	53	56	54	55	53	51	50	51	52	−0.8 (−1, −0.4)
Phenoxymethylpenicillin/100 antibiotic prescriptions	60	54	55	57	56	62	66	67	71	3 (2,3)
Fever										
Episodes/1000 children	86	94	87	86	83	88	77	85	89	−1 (−3, −1)
Antibiotic prescriptions/100 episodes	18	21	19	20	18	17	16	15	15	−1 (−1, −1)
Phenoxymethylpenicillin/100 antibiotic prescriptions	55	50	51	53	55	57	59	60	63	2 (2,2)
Tonsillitis										
Episodes/1000 children	38	49	41	40	38	39	35	30	31	−2(−3, −2)
Antibiotic prescriptions/100 episodes	71	72	71	72	71	71	69	68	69	−0.5 (−0.8, −0.3)
Phenoxymethylpenicillin/100 antibiotic prescriptions	74	71	69	71	72	75	78	79	81	2 (1,2)
Bronchitis										
Episodes/1000 children	37	38	31	35	40	49	44	36	29	−0.1 (−2,2)
Antibiotic prescriptions/100 episodes	26	37	31	31	25	23	20	19	19	−3 (−3, −2)
Phenoxymethylpenicillin/100 antibiotic prescriptions	39	29	33	36	38	44	49	47	47	3 (2,4)
Pneumonia										
Episodes/1000 children	7	13	9	7	6	6	6	4	4	−1 (−2, −1)
Antibiotic prescriptions/100 episodes	69	71	70	71	68	70	68	66	64	−1 (−1,0)
Phenoxymethylpenicillin/100 antibiotic prescriptions	44	32	43	44	47	50	48	54	57	3 (2,4)

### Episodes

A child with an RTI may need repeated healthcare contacts. By combining contacts in episodes, we get information on the course of illness, repeated contacts and when antibiotics are prescribed. An episode starts the day a child receives a RTI diagnosis if the child has not received a RTI diagnosis within the previous 30 days. Contacts <30 days after an RTI contact are defined as part of the same RTI episode. The period of 30 days was chosen on the basis of earlier studies of RTI episodes.^18,19^ The follow-up period ended at 90 days; a period chosen to define the duration of episodes in adult populations, and made the numbers comparable.

During an episode, a child might receive several RTI diagnoses. The diagnose most likely to lead to antibiotic prescription is defined as the main diagnose in the episode (Table S1).

### Antibiotics

Following the ATC classification, we included the J01 group of antibiotics for oral use, excluding methenamine, mecillinam, trimethoprim and nitrofurantoin as they are exclusively prescribed for urinary tract infections in Norway. Antibiotic classes were grouped as phenoxymethylpenicillin (J01CE02), other penicillins (J01C excluding J01CE02), macrolides (J01F) and others (J01A, J01DB, J01EE, J01M, J01X).

Antibiotic prescriptions dispensed within 7 days of a consultation were allocated to the episode and included in the dataset for analysis. This 7-day window was chosen to capture ‘wait-and-see’ prescriptions; however, 96% of prescriptions were dispensed within 2 days during the follow up.

### Statistical analysis

The material is described with means and proportions calculated for each year and for the entire study period. The rate of annual RTI episodes was calculated by dividing the number of episodes on the number of children in the defined age group for each year. To be able to compare the rates over years, independent of fluctuations in the population, the rates were standardized by age and gender compared to the population in 2012.

The study mean RTI episode rates are the mean of the yearly episode rates in the period 2012 to 2019. The prescription rate is the number of episodes resulting in an antibiotic prescription collected from a Norwegian pharmacy, divided by the total number of episodes. The relative prescription rates were calculated for different types of antibiotic. The phenoxymetylpenicillin rate is the proportion of phenoxymetylpenicillin of the total amount of antibiotics prescribed.

We used linear regression to analyse trends in RTI episodes and the number of contacts in each episode over years. Linear regression of RTI episodes over year was used for each group of children: grouped in ages 1–5 years, gender and for the different RTI diagnoses. We analysed the total RTI episodes as well as all RTI diagnoses individually. The seven RTI diagnoses with the highest total amount of antibiotics prescribed were presented in detail. Linear regression over 1 year was also used to analyse yearly prescription rates and the proportion of different types of antibiotic prescribed. Coefficients from the regressions are presented as mean yearly change with 95% CI. In addition, a negative binomial regression was conducted on episode counts over the years using the yearly population of children aged 1–5 as an offset.

Populations weighted linear regression models are easy to interpret reliable models to model time trends and results in reliable results for nationwide data and using the same analytical approach as our previous report on adult prescription in the same setting maximize comparability.^19,20^

The calculations are made using Stata v.17.0 (StataCorp LLC). The significance level was set to 0.05. Means are presented with standard deviation (SD).

## Results

### RTI episodes

The dataset contains 578 965 children between 1 and 5 years each year in the period 2012 −2019 (Table 1), with 54% of the children being boys.

In total, the children had 3 126 818 contacts with RTI diagnoses between 2012 and 2019, amounting to 2 003 904 RTI episodes by our definition, giving an overall rate of 811 RTI episodes/1000 children per year. The boys had an RTI rate of 846/1000 and the girls 774/1000. Mean age at first contact was 1.9 years (SD 1.3).

A little less than half of the episodes contained only one contact (46%). Repeated contacts in an episode were more common for the youngest children. One-year-olds had 2.4 contacts per episode (SD 1.9), and 5 year olds had 1.9 contacts per episode (SD 1.5). There were no gender differences in mean number of contacts [2.3 (SD 1.8) for boys compared with 2.2 (SD 1.7) for girls]. The differences in the mean number of contacts in each episode for the seven RTI diagnoses with the highest antibiotic prescription rates were small and non-significant, ranging from 2.4 (SD 1.8) for R81 pneumonia, to 2.2 (SD1.7) for R05 cough. There was no annual change in the number of contacts within each episode in the period 2012–2019.

Upper RTIs (R74 URTI) was the most used diagnosis, followed by R05 cough and H71 otitis. There were minor differences in gender distribution between diagnoses (Table S2). The RTI episode rate was much higher for the youngest children, 1279 episodes/1000 children for 1 year- olds and 458 episodes/1000 children for 5 year olds. Children aged 1 and 2 years accounted for 56% of the episodes (Figure 1).

Total RTI episode rate was 941 episodes/1000 children in 2012, reduced by 17% to 2019 when there were 777 episodes/1000 children. Mean annual change was −18 (95%CI −33, −4). Negative binomial regression showed a 2.8% reduction in episode rates per year.

The reduction differed between diagnoses. The largest relative reduction from 2012 to 2019 was seen for the diagnosis R81 pneumonia (reduced from 13/1000 to 4/1000), while R74 URTI was used slightly more in 2019. R04 Cough had the largest absolute reduction in episode rate, from 136/1000 to 71/1000.

### Antibiotic prescriptions

The total prescription rate was reduced from 28% in 2012 to 19% in 2019. The largest annual reduction in prescription rate was observed from 2012 (28%) to 2013 (24%). The change in prescription rate differed between the years (Table 2). Only 40% of antibiotic prescriptions were prescribed at the first contact in an episode. Two diagnoses accounted for more than half of the prescriptions, H71 Otitis (26% of total, 119 900 prescriptions) and R74 URTI (25% of total, 113 882 prescriptions) (Figure 2). The decrease in antibiotic prescription rate was present for almost all the most used RTI diagnoses, but the size of the change differed. The largest reduction in antibiotic prescription rate from 2012 to 2019 was seen for R78 bronchitis/bronchiolitis from 37% to 19% (Table 2). Three diagnoses had particularly high total prescription rates in the study period: H71 otitis (53%), R72 + 76 tonsillitis (71%) and R81 pneumonia (69%). The antibiotic prescription rate for H71 otitis decreased from 56% in 2012 to 52% 2019, but because fewer children received the diagnosis the total amount of expedited antibiotic prescriptions for otitis was reduced by 42%.

Phenoxymethylpenicillin was the most used antibiotic in the treatment of RTIs in preschool children, mean proportion 57% (Table 3). In addition to a decrease in the total amount of antibiotics prescribed to this group of patients, there was a shift in the type of antibiotics prescribed. Although the number of prescriptions was reduced from 2012 to 2019 for all types of antibiotics, phenoxymethylpenicillin (ATC code J01CE02) increased in proportion of the total prescribed antibiotics, 50% in 2012 versus 68% in 2019, respectively. Macrolides had a steadily reducing rate of prescriptions, from 18 519 (23% of total) prescriptions in 2012 to 4933 (12% of total) in 2019 (Table 3). The diagnoses with the largest decrease in macrolide rate from 2012 to 2019 were R74 URTI (47% reduction), R81 pneumonia (46% reduction), R78 bronchitis/bronchiolitis (45% reduction) and H71 otitis (43% reduction).

**Table 3.** Dispensed antibiotic prescriptions for acute RTIs in preschool children 2012–2019

	Total	2012	2013	2014	2015	2016	2017	2018	2019	Mean annual change (95%CI)
Total episodes with dispensed antibiotic prescriptions	456 476	81 735	63 989	66 072	56 424	55 003	47 904	42 710	42 639	
Penicillin’s J01CA	369 830	61 311	50 736	52 625	45 807	45 528	40 335	36 774	36 714	
Percentage of total antibiotic prescriptions	81	75	79	80	81	83	84	86	86	1.5 (1.2, 1.8)
Phenoxymethylpenicillin CE02	262 451	40 980	34 163	35 806	31 685	32 961	30 331	27 639	28 886	
Percentage of total antibiotic prescriptions	57	50	54	56	63	60	63	65	68	2.4 (1.6, 3.1)
Macrolides J01FA	75 334	18 519	11 602	11 669	9 139	8 140	6 396	4 946	4 933	
Percentage of total antibiotic prescriptions	17	23	18	17	16	15	14	12	12	−1.4 (−1.9, −0.9)
Others (J01AA, J01DB, J01EE, J01MA, J01XX)	11 302	1 905	1 651	1 778	1 478	1 335	1 173	990	992	
Percentage of total antibiotic prescriptions	2	2	3	3	3	2	2	2	2	−0.1 (−0.3, 0.1)

## Discussion

### Main findings

The observed reduction in the total amount of antibiotics prescribed to preschool children is explained by both a reduction in the RTI episode rate (17% decrease from 2012 to 2019), and in the antibiotic prescription rate per episode in the period (32% decrease from 2012 to 2019*).* There was also a relative increase in the proportion of phenoxymethylpenicillin prescribed.

For some diagnoses, such as R78 bronchitis/bronchiolitis, there was a significant reduction in the antibiotic prescription rate, for others, sduch as H71 otitis, the prescription rate barely changed, while the episode rate decreased considerably.

### Comparison with other studies

#### Change in health-seeking behaviour

The reduction in episode rate is largest for pneumonia and cough, and most of the change happened between 2012 and 2013, coinciding with an outbreak of *Mycoplasma Pneumonia* in 2012.^21^ Younger children tend to receive more symptom diagnoses, such as cough, fever and URTI. This may be due to the difficulties clinicians face when examining very young patients, as well as the nonspecific nature of respiratory infections in preschool-aged children.^22^ The mean contact rate is also notably high, indicating that revisits and repeated evaluations are common within this age group. The introduction of pneumococcal vaccine in 2009, reduced the incidence of pneumonia and otitis in children under 2 years.^23–25^ With high levels of vaccination in Norway it is unlikely that the vaccine can explain the observed reduction in episode rate from 2012 to 2019. This may indicate that increased public awareness on antibiotic stewardship have changed how parents manage their sick child. The experience of not being prescribed antibiotics on a doctor’s visit, but instead advise on how to evaluate the child’s illness and treat self-limiting respiratory infections at home with symptomatic relief of pain and fever, may have enabled many parents to manage without contacting the doctor.

Otitis is the RTI diagnosis that generates the greatest number of antibiotic prescriptions. It is interesting that the antibiotic prescription rate for otitis is almost unchanged from 2012 to 2019, and that the reduction in antibiotic treatment correlate with a change in how often parents bring their child with ear symptoms to the doctor. Guidelines recommend antibiotics only in specific cases, and we were surprised by the consistent high rate of antibiotic prescriptions for otitis. One possible explanation for the stable prescription rate is that the reduction in episode rate has led to a population of children with more severe symptoms, resulting in a higher percentage who meet the criteria for antibiotics. This may indicate that parents have become more confident at self-managing children with ear symptoms, and when to seek medical attention, but this needs further investigation in studies of qualitative design.

Another interpretation is that there still is room for improvement in diagnosis and adherence to guidelines in the choice of treatment for uncomplicated otitis. There were no differences between the children in 2012 and 2019 regarding contacts per episode or the total number of otitis episodes per child, and it is unlikely that a higher percentage had recurring or chronic ear infections. As the guidelines differ for the youngest children, we performed a sensitivity analysis, using age defined in months rather than years for greater precision. The prescription rate remained ∼50% for all ages, and there was little change in the prescription rate during the study period (Table S3). The guidelines recommend liberal use of antibiotics to children with chronic or repeated ear infections.^6^ When comparing children with otitis episodes in 2012 and 2019, we found no difference between the two.

### Reduction in total amount of antibiotics and prescription rates and increased proportion of phenoxymethylpenicillin

The decrease in antibiotic prescribing between 2012 and 2019, corresponds with previous Norwegian studies and studies of adults from the same period.^26,27^ Other countries have experienced a similar reduction.^28–32^ Studies from high-income countries show a similar reduction in antibiotic prescription rates after antibiotic stewardship campaigns.^33,34^ The results from this study can be interpreted as a combination of a more cautious antibiotic prescribing practice, and less demand for antibiotics from the public: both targets for national campaigns.

The largest reduction is seen for diagnoses with a probable viral aetiology, like bronchitis/bronchiolitis, while diagnoses like otitis and tonsillitis have an almost unchanged prescription rate. This may indicate that the first effect of stewardship campaigns is to reduce the use of antibiotics in the treatment of infections with a high likelihood of viral aetiology, such as bronchitis/bronchiolitis and common colds. Even a moderate reduction in the prescription rate for URTI has a large impact, since it is the most used RTI diagnosis in this age group. The prescription rates for self -limiting RTI’s of probable viral aetiology, like URTI, cough and bronchitis/bronchiolitis, are still concerningly high, and there is room for greater adherence to guidelines. Norwegian general practitioners have access to their antibiotic prescription numbers, and it is hoped that this will increase awareness and increase adherence to guidelines. The increased use of phenoxymethylpenicillin in the period is in accordance with the guidelines and the national strategy against AMR.^5,6^

The use of phenoxymethylpenicillin is well established and experienced as safe, and the use of narrow spectrum antibiotics less resistance driving in a population than other antibiotics,^35^ although a recent study from Sweden claims that phenoxymethylpenicillin changes the gut microbiome more than previously understood.^36^

The official guidelines for choice of antibiotics are similar in the Nordic countries,^37,38^ but in Denmark slightly more of the less ill-tasting Amoxicillin (J01CA04) is prescribed to the youngest preschool children with RTI. The unpleasant taste of phenoxymethylpenicillin makes it difficult to administer to small children and may influence the parents demand for antibiotic treatment.^39^

### Strengths and limitations

The main strength of this study is the size and quality of the material, giving data on the entire population in the period analysed.

Registry data are collected for administrative use, and there are severe limitations in the use of registry data in research. By combining information from several registries, it is possible to follow a child through repeated healthcare contacts, but the interpretation of the findings is limited to the available numbers. We have no information on the background for choosing a diagnosis, or objective parameters to assess whether correct treatment was administered.

Small children have more frequent RTIs than adults, perhaps making the 90-day follow-up period long. However, we chose to keep it, making the study comparable to studies of adults.

Another limitation is that our analyses did not account for seasonality or within-child clustering, but for nationwide yearly trends we believe this to have a limited impact.

The overall result is also skewed because of a mycoplasma outbreak in 2012. There will be naturally occurring outbreaks of different RTIs among preschool children, and years with high occurrence of mycoplasma, influenza or whooping cough can cause large fluctuations in episode rates and antibiotic prescription rates. The numbers and the size of reduction in prescription rates over a shorter period must therefore be interpreted with caution.

Owing to missing data for 2018 and 2019, we were only able to calculate age in months for the period from 2012 to 2017 in the sensitivity analysis of otitis.

### Implications

The study shows that it is possible to reduce the prescription of antibiotics also in a country with initially low prescription rates. A large amount of total antibiotics was prescribed to RTI’s with a predominantly viral origin and may represent inappropriate treatment. The prescription rate for otitis was surprisingly high and stable, and treatment may not be in accordance with prescription guidelines. On the basis of our study, the treatment of otitis in children in general practice, is an area of interest for future stewardship campaigns directed towards GPs.

Some of the total reduction in antibiotic prescription correlates to a lower rate of RTI episodes. It is important to increase the health literacy in the population, enabling parents to better evaluate and care for their sick child.

Recent reports show a large increase in antibiotic prescriptions in the post-pandemic years.^2^ The ESAC report of 2023 recommend continued efforts to reduce antibiotic prescriptions in all European countries to combat AMR.^3^

## Supplementary Material

dlaf231_Supplementary_Data

## Supplementary data

Tables S1–S3 and Figures S1 and S2 are available as Supplementary data at *JAC-AMR* Online.
