# Supplementary material for: Antibiotic prescriptions to preschool children with respiratory tract infections in primary healthcare
Source: JAC Antimicrob Resist. 2026 Jan 8;8(1):dlaf231. doi: 10.1093/jacamr/dlaf231 (PMC12780767; doi:10.1093/jacamr/dlaf231)
Supplement: dlaf231_Supplementary_Data [file dlaf231_supplementary_data.docx]

Supplementary data:

**Figure S1: Flowchart of population in material, children treated in primary health care from 2012 - 2019**

*F*

**Figure S2: Trends in respiratory tract infection (RTI) episodes, antibiotic rates and phenoxymethylpenicillin rates for children 1-5 years old, treated in primary health care from 2012 - 2019**

**Table S1:** ICPC-2 Diagnoses included in the dataset by antibiotic prescriptions rate.

| Diagnoses from lowest to highest prescription rate | |
| --- | --- |
| 1 "R23 Voice symptom/complaint" | |
| 2 "R04 Breathing problem" | |
| 3 "R08 Nose symptom/complaint" |  |
| 4 "R77 Laryngitis/tracheitis acute" | |
| 5 "R25 Sputum/phlegm abnormal" |  |
| 6 "R03 Wheezing" |  |
| 7 "R07 Sneezing/Nasal congestion" | |
| 8 "R01 Pain respiratory system" | |
| 9 "A76 Viral exanthema + A77 Viral disease other" | |
| 10 "R02 Dyspnea" |  |
| 11 "R80 Influenza" |  |
| 12 "R05 Cough" |  |
| 13 "R29 Respiratory symptom/complaint " | |
| 14 "R74 Upper respiratory infection " |  |
| 15 "A03 Fever" |  |
| 16 "H01 Ear pain/earache" | |
| 17 "R83 Respiratory infection other" | |
| 18"R09 Sinus symptoms" | |
| 19 "R78 Acute bronchitis/bronchiolitis" | |
| 20 " R21 Throat symptom/complaint" | |
| 21 "R71 Whooping cough" | |
| 22 "H71 Acute otitis media" | |
| 23 "R75 Sinusitis acute/chronic" | |
| 24 "R81 Pneumonia" | |
| 25 "R72 Strep throat +R76 Tonsillitis acute" | |

**Table S2:** Gender distribution for different respiratory tract diagnoses (n =2 003 904 episodes)

| Diagnosis | |  |  |  |  |
| --- | --- | --- | --- | --- | --- |
|  |  |  | **Male (%)** | **Female** | **Total** |
| 1 "R23 Voice symptom/complaint" | | | 1 025(60) | 685 | 1 710 |
| 2 "R04 Breathing problem" | | | 7 039(57) | 5 324 | 12 363 |
| 3 "R08 Nose symptom/complaint" | | | 3 607(56) | 2 856 | 6 463 |
| 4 "R77 Laryngitis/tracheitis acute" | | | 41 067(64) | 22 946 | 64 013 |
| 5 "R25 Sputum/phlegm abnormal" | | | 60(64) | 34 | 94 |
| 6 "R03 Wheezing" | |  | 452(63) | 266 | 718 |
| 7 "R07 Sneezing/Nasal congestion" | | | 6 466(57) | 4 887 | 11 353 |
| 8 "R01 Pain respiratory system" | | | 26(62) | 16 | 42 |
| 9 "A76 Viral exanthema + A77 Viral disease" | | | 56 918(53) | 50 065 | 106 983 |
| 10 "R02 Dyspnea" | |  | 8 136(62) | 4 928 | 13 064 |
| 11 "R80 Influenza" | |  | 12 570(52) | 11 640 | 24 210 |
| 12 "R05 Cough" | |  | 121 359(52) | 110 896 | 232 255 |
| 13 "R29 Respiratory symptom/complaint " | | | 8 410(57) | 6 448 | 14 858 |
| 14 "R74 Upper respiratory inf." | | | 374 274(53) | 332 188 | 706 462 |
| 15 "A03 Fever" | |  | 110 102(52) | 102 901 | 213 003 |
| 16 "H01 Ear pain/earache" | | | 17 261(51) | 16 890 | 34 151 |
| 17 "R83 Respiratory infection other" | | | 48 229(54) | 41 954 | 90 183 |
| 18"R09 Sinus symptoms" | | | 109(56) | 87 | 196 |
| 19 "R78 Acute bronchitis" | | | 50 753(55) | 41 322 | 92 075 |
| 20 " R21 Throat symptom/complaint" | | | 21 320(53) | 18 811 | 40 131 |
| 21 "R71 Whooping cough" | | | 605(52) | 556 | 1 161 |
| 22 "H71 Acute otitis media" | | | 122 195(54) | 104 084 | 226 279 |
| 23 "R75 Sinusitis acute/chronic" | | | 565(51) | 539 | 1 104 |
| 24 "R81 Pneumonia" | | | 9 019(53) | 7 945 | 16 964 |
| 25 "R72 Strep throat +R76 Tonsillitis acute" | | | 51 759(55) | 42 310 | 94 069 |
|  |  |  |  |  |  |
| Total | | | 1 073 326(54) | 930 578 | 2 003 904 |

**Table S3:** Antibiotic prescription rate for otitis, age defined by months.

| Otitis | 2012 | 2013 | 2014 | 2015 | 2016 | 2017 | Sum |
| --- | --- | --- | --- | --- | --- | --- | --- |
| 12 months |  |  |  |  |  |  |  |
| Antibiotic prescriptions | 7 070 | 5 877 | 5 836 | 4 951 | 4 991 | 4 259 | 32 984 |
| Otitis episodes | 11 889 | 10 059 | 9 750 | 8 831 | 8 813 | 7 821 | 57 163 |
| % | 59 | 58 | 60 | 56 | 57 | 54 | 58 |
|  |  |  |  |  |  |  |  |
| 24 months |  |  |  |  |  |  |  |
| Antibiotic prescriptions | 4 827 | 3 845 | 3 941 | 3 305 | 3 102 | 2 802 | 21 822 |
| Otitis episodes | 8 528 | 7 083 | 7 199 | 6 352 | 6 120 | 5 575 | 40 857 |
| % | 57 | 54 | 55 | 52 | 51 | 50 | 53 |
|  |  |  |  |  |  |  |  |
| 36 months |  |  |  |  |  |  |  |
| Antibiotic prescriptions | 3 333 | 2 822 | 2 811 | 2 497 | 2 214 | 2 062 | 15 739 |
| Otitis episodes | 6 340 | 5 467 | 5 414 | 4 972 | 4 712 | 4 378 | 31 283 |
| % | 53 | 52 | 52 | 50 | 47 | 47 | 50 |
|  |  |  |  |  |  |  |  |
| 48 months |  |  |  |  |  |  |  |
| Antibiotic prescriptions | 2 596 | 2 295 | 2 227 | 1 989 | 1 854 | 1 604 | 12 565 |
| Otitis episodes | 4 946 | 4 585 | 4 537 | 4 075 | 3 997 | 3 640 | 25 780 |
| % | 53 | 50 | 49 | 49 | 46 | 44 | 49 |
|  |  |  |  |  |  |  |  |
| 60 months |  |  |  |  |  |  |  |
| Antibiotic prescriptions | 1 973 | 1 682 | 1 709 | 1 551 | 1 530 | 1 363 | 9 808 |
| Otitis episodes | 3 786 | 3 416 | 3 491 | 3 316 | 3 136 | 2 905 | 20 050 |
| % | 52 | 49 | 49 | 47 | 49 | 47 | 49 |
|  |  |  |  |  |  |  |  |
| Total |  |  |  |  |  |  |  |
| Antibiotic prescriptions | 19 799 | 16 521 | 16 524 | 14 293 | 13 691 | 12 090 | 92 918 |
| Otitis episodes | 35 489 | 30 610 | 30 391 | 27 546 | 26778 | 24 319 | 175 133 |
| % | 56 | 54 | 54 | 52 | 51 | 50 | 53 |

F
